# Somatostatin receptor expression in Merkel cell carcinoma as target for molecular imaging

**DOI:** 10.1186/1471-2407-14-268

**Published:** 2014-04-17

**Authors:** Kristina Buder, Constantin Lapa, Michael C Kreissl, Andreas Schirbel, Ken Herrmann, Alexander Schnack, Eva-Bettina Bröcker, Matthias Goebeler, Andreas K Buck, Jürgen C Becker

**Affiliations:** 1 Department of Dermatology, Venereology and Allergology, University Hospital Würzburg, Josef-Schneider-Strasse 2, 97080 Würzburg, Germany; 2 Comprehensive Cancer Center Mainfranken, University Hospital Würzburg, Josef-Schneider-Strasse 6, 97080 Würzburg, Germany; 3 Department of Nuclear Medicine, University Hospital Würzburg, Oberdürrbacher Strasse 6, 97080 Würzburg, Germany; 4 Department of Nuclear Medicine, Central Hospital of Augsburg, Stenglinstr.2, 86156 Augsburg, Germany; 5 Department of Radiology, University Hospital Würzburg, Oberdürrbacher Strasse 6, 97080 Würzburg, Germany; 6 Department of General Dermatology, Medical University Graz, Auenbrugger Platz 1, 8036 Graz, Austria

**Keywords:** Merkel cell carcinoma, Molecular imaging, Somatostatin receptor expression, Positron emission tomography

## Abstract

**Background:**

Merkel cell carcinoma (MCC) is a rare cutaneous neoplasm with increasing incidence, aggressive behavior and poor prognosis. Somatostatin receptors (SSTR) are expressed in MCC and represent a potential target for both imaging and treatment.

**Methods:**

To non-invasively assess SSTR expression in MCC using PET and the radiotracers [^68^Ga]DOTA-D-Phe^1^-Tyr^3^-octreotide (DOTATOC) or -octreotate (DOTATATE) as surrogate for tumor burden. In 24 patients with histologically proven MCC SSTR-PET was performed and compared to results of computed tomography (CT).

**Results:**

SSTR-PET detected primary and metastatic MCC lesions. On a patient-based analysis, sensitivity of SSTR-PET was 73% for nodal metastases, 100% for bone, and 67% for soft-tissue metastases, respectively. Notably, brain metastases were initially detected by SSTR-PET in 2 patients, whereas liver and lung metastases were diagnosed exclusively by CT. SSTR-PET showed concordance to CT results in 20 out of 24 patients. Four patients (17%) were up-staged due to SSTR-PET and patient management was changed in 3 patients (13%).

**Conclusion:**

SSTR-PET showed high sensitivity for imaging bone, soft tissue and brain metastases, and particularly in combination with CT had a significant impact on clinical stage and patient management.

## Background

Merkel cell carcinoma (MCC) is a rare, highly aggressive, viral associated cutaneous neoplasm with neuroendocrine characteristics [1,2]. Indeed, it is characterized by expression of neuroendocrine markers including somatostatin receptors (SSTR) [3,4]. Five-year survival rates are as low as 66% for stage I, 51% for stage II, 39% and 18% for stage III and IV, respectively [5]. While a standardized staging system has been introduced with the 7^th^ edition of the AJCC staging manual [6,7], the definite staging algorithm for MCC remains to be established. Current imaging procedures for patients with clinical stage I/II disease include ultrasonography of regional lymph nodes and the abdomen as well as a chest X-ray. A sentinel lymph node biopsy (SLNB) is recommended for all patients with no evidence of lymph node or distant metastasis [8-11]. Contrast-enhanced computed tomography (CT) is generally performed in patients with clinical stage III/IV disease. Functional or molecular imaging modalities such as ^18^ F-fluorodeoxyglucose positron emission tomography (FDG-PET) are increasingly used [12-17].

In analogy to neuroendocrine tumors (NET), SSTR expression may be used for staging [18]. ^68^Ga-labeled 1,4,7,10-tetraazacyclo-dodecane-N,N’,N”,N”-tetraaceticacid D-Phe^1^-Tyr^3^-octreotide (^68^Ga-DOTATOC) and Tyr^3^-octreotate (^68^Ga-DOTATATE) are somatostatin analogs with high affinity to SSTR subtype 2 suitable for PET imaging, thereby offering superior spatial resolution [19]. Radiotracer uptake has been shown to correlate with expression of SSTR 2 in NET and MCC [3,20,21]. SSTR-PET is more sensitive and accurate for tumor detection than respective scintigraphic techniques [22]. SSTR-PET has been claimed to be beneficial compared to conventional imaging and FDG-PET in selected patients with MCC [23,24].

The aim of this study was to assess the impact of non-invasive characterization of SSTR expression in MCC on tumor staging, as compared to conventional staging by CT and to explore its suitability as molecular target for treatment of metastatic MCC.

## Methods

### Patients

In 24 patients with histologically confirmed MCC, SSTR-PET was performed. In a sub-cohort of 8 patients, repetitive imaging was performed. The cohort included 16 male and 8 female patients with a mean age of 68 years at inclusion (range 44–81). At the initial diagnosis, 6 patients had stage I disease, 5 patients were stage II, 10 patients were stage III and 3 patients were stage IV. Two patients had a history of secondary malignancy in complete remission. The median follow-up was 36 months (range 18–57 months).

Due to the retrospective nature of our study, the requirement for approval has been waived by the local ethics committee of the University of Würzburg. Since 2009, the German federal law accepts the use of the radiotracer ^68^Ga-DOTATATE under conditions of the pharmaceutical law. Before that time point, the use of ^68^Ga-DOTATATE was approved on a compassionate use base. Nevertheless, in all of our patients, informed consent was obtained prior to the imaging procedure.

### Study design

In this retrospective study, imaging studies of consecutive patients with MCC examined between 05/2008 and 09/2011 were analyzed. SSTR-PET was performed in the clinical routine on a compassionate use basis; informed consent for the imaging procedures was obtained. It is a retrospective analysis of single institutional data. Patient’s consent was obtained for publication of illustrations including photos. CT of the thorax and abdomen served as reference. SSTR-PET and CT data were acquired within a mean interval of 12.5 days (range, 0–45). In between, no surgery or systemic treatment was performed. Head-neck MRI was performed if clinically indicated. In 2010, an integrated PET/CT scanner was introduced, enabling combined acquisition of PET and CT data in 5 patients. In all patients, SSTR-PET and CT data were documented as separate files, enabling separate evaluation of PET and CT data.

Eight patients were re-staged by SSTR-PET after first-line chemotherapy with liposomal doxorubicin or cisplatin-based polychemotherapy.

### Somatostatin receptor PET

^68^Ga-DOTATOC/^68^Ga-DOTATATE was prepared using a modification of the method described by Breeman et al. [25] using a radiotracer synthesis module (Scintomics, Fürstenfeldbruck, Germany). PET-scans were acquired using a Siemens PET scanner (ECAT Exact 47, Siemens Health Care, Erlangen, Germany). Acquisition started 30–45 minutes after intravenous injection of ^68^Ga-DOTATOC (116 ± 46 MBq).

SSTR-PET/CT was performed on a dedicated scanner (Siemens Biograph mCT 64, Siemens, Knoxville, USA) 40–60 min after injection of ^68^Ga-DOTATATE (122 ± 52 MBq).

Standardized uptake values (SUV) were calculated by assigning spherical volumes of interest of 1.5 cm diameter including foci of increased tracer uptake. In addition, mean and maximum SUV values (SUV_max_, SUV_mean_) were calculated. A 1.5 cm spherical volume of interest was drawn also to the center of the right lobe of the liver to determine the SUV_mean_ liver as reference for background activity.

### CT

Spiral CT of the chest and abdomen was performed using a 16-slice multi-detector CT (Siemens Sensation 16, Siemens Healthcare) and intravenous contrast. If clinically indicated the head, neck or lower extremities were also included. In two stage I patients SPECT-CT images were obtained during SLN detection. In one stage I patient MRI was performed instead of CT.

### Image interpretation and data analysis

All images were reviewed by two experienced nuclear medicine physicians (C.L. and M.C.K.) and an experienced radiologist (Al.S.), who were blinded to clinical data. Analyses were performed on a patient and on a lesion basis. Lesion-based analysis was restricted to a maximum of 10 lesions per organ.

## Results

### Staging results of SSTR-PET and CT; patient-based analysis

SSTR-PET was able to detect MCC lesions in all patients with primary and/or metastatic disease with mild to moderate tracer uptake. Concordance of CT and SSTR-PET was observed in 20 of 24 patients (83%) in a patient-based analysis; change of management due to SSTR-PET occurred in 4/24 (17%) patients (Table 1). On a patient basis, CT and SSTR-PET were concordant regarding overall tumor stage in all but one patient.

**Table 1:** Tumor stage of MCC patients, as assessed by SSTR-PET and CT

**No.**	**SSTR-PET**	**CT**	**Up-staging by SSTR-PET**	**Change of the therapeutic management due to SSTR-PET**
1	IA*	IA*	No	-
2	IA*	IA*	No	-
3	IA	IA	No	-
4	IA*	IA*	No	-
5	IA*	IA*	No	-
6	IA*	IA*	No	Parotid surgery (Warthin tumor)
7	IIIA*	IIIA*	No	-
8	IIIB	IIIB	No	-
9	IIIB	IIIB	No	-
10	IIIB	IIIB	No	-
11	IIIB	IIIB*	No	-
12	IIIB*	IIIB*	No	-
13	IIIB*	IIIB*	No	-
14	IV	IV	No	Bisphosphonate Tx (bone mets)
15	IIIB	IIC	Yes	Lymph node exstirpation (positive)
16	IV	IV	Yes	-
17	IV	IV	No	Bisphosphonate Tx (bone mets)
18	IV	IV	No	-
19	IV	IV	No	-
20	IV	IV	Yes	-
21	IV	IV	No	-
22	IV	IV	Yes	-
23	IV	IV	No	Bisphosphonate Tx (bone mets)
24	IV	IV	No	-

Clinical stage as indicated by SSTR-PET and CT. Asterisks (*) indicate patients free of disease. Upstaging by SSTR-PET as compared to conventional imaging. Change of management due to SSTR-PET was seen in 5 patients.

**Figure 1:**
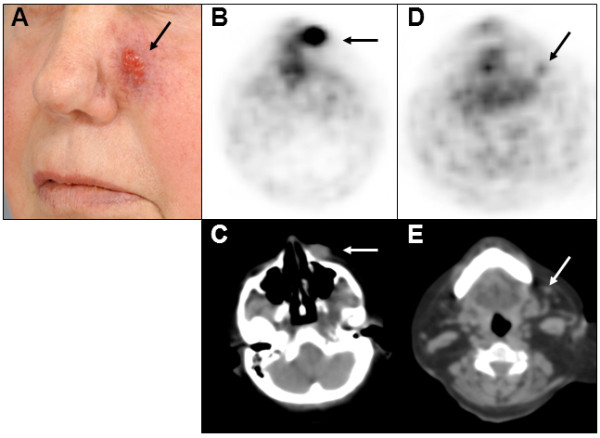
**Primary MCC and lymph node metastasis visualized by SSTR-PET.** A female patient (no. 15) presented with a primary MCC on her left cheek **(A)**, which was visualized by both SSTR-PET **(B)** and CT **(C)**. Additionally, SSTR-PET revealed increased tracer uptake **(D)** in a non-enlarged lymph node **(E)**. After resection, a 7 x 5 mm lymph node metastasis was histologically confirmed.

Three patients received SSTR-PET when the primary tumor was present. Patient 3 had a T1 tumor, patient 15 an infiltrating T4 tumor (Figure 1) and patient 22 a T2 tumor with satellite metastases in an area of 6 × 8 cm. All primary tumors showed focally increased tracer uptake.

When compared to CT, lymph node metastases were correctly identified by SSTR-PET in 8 of 11 patients (sensitivity 73%; Table 2A). One patient was correctly up-staged to stage IIIB (pN1b) by SSTR-PET but not by CT. In 6/6 patients with clinical stage I disease both SSTR-PET and CT indicated N0 disease. SLNB was negative in these 6 patients, confirming absence of nodal involvement.

**Table 2:** Patient- and lesion-based analysis of SSTR-PET and CT

**Metastatic sites**	**A) Patient-based assessment**			**B) Lesion-based assessment**		
	**SSTR-PET**	**CT**	**SSTR-PET sensitivity/specificity/accuracy**	**SSTR-PET**	**CT**	**SSTR-PET sensitivity**
**Lymph node**	8	11	73%/ 92%/ 83%	62	105	59%
**Lung**	0	2	0%/ 100%/ 95%	0	6	0%
**Pleura**	1	2	50%/ 100%/ 95%	1	2	50%
**Pancreas**	1	1	n/a	1	1	n/a
**Bone**	5	2	100%/ 83%/ 85%	35	20	100%
**Soft tissue**	5	3	67%/ 90%/ 86%	8	2	100%
**Liver**	0	5	0%/ 100%/ 80%	0	33	0%

(A) Patient-based and (B) lesion-based analysis of SSTR-PET and CT results regarding detection of lymph node and distant metastases from Merkel cell carcinoma.

**Figure 2:**
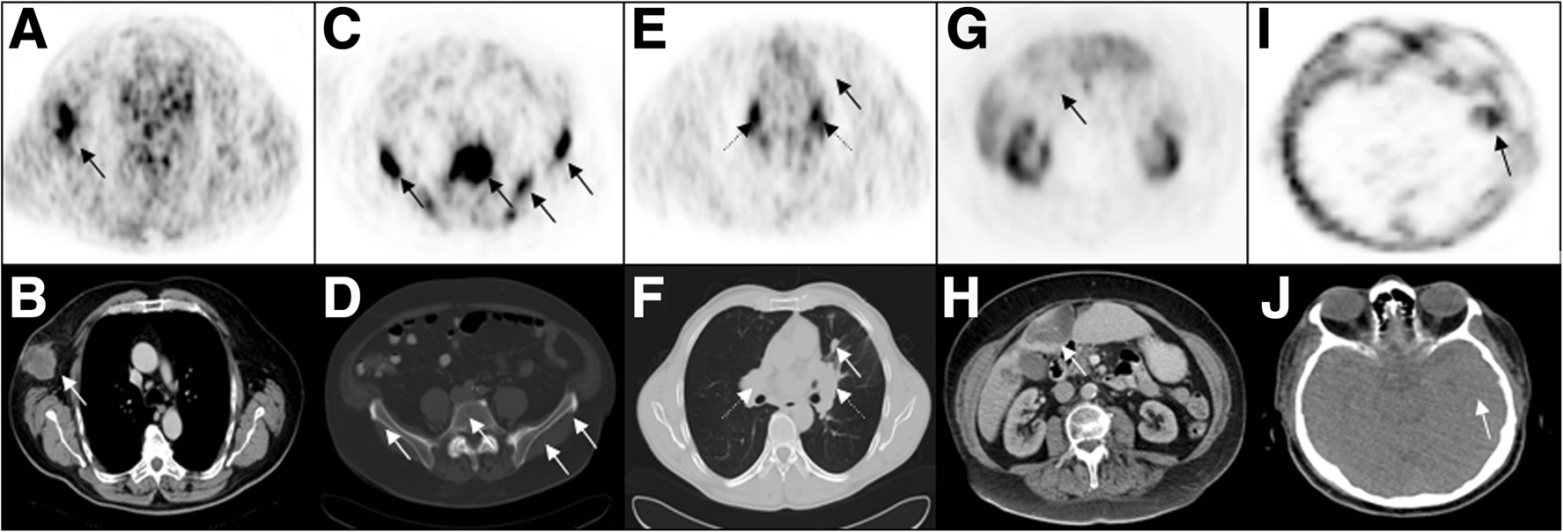
**Typical example of SSTR-positive metastases visualized by SSTR-PET.** Example of SSTR-positive MCC metastases which were displayed by SSTR-PET imaging. Corresponding CT sections are shown below the transaxial PET views. Whereas lymph node metastases can be visualized with comparable sensitivity (arrows, **A, B, E, F**), SSTR-PET is more sensitive for detecting bone and brain metastases as compared to CT **(C, D, I, J)**. On the other hand, lung and liver metastases (arrows, **E, F**) are detected with lower sensitivity by SSTR-PET.

SSTR-PET correctly detected distant metastatic disease in 10/10 patients. However, there were clinically significant organ-specific differences in the number of metastatic sites as compared to CT (Table 2A, Figure 2). Patient-based analysis for different metastatic sites revealed 5 patients with bone metastases by SSTR-PET versus 2 patients identified by CT (Table 2A). Soft-tissue metastases were detected by SSTR-PET with a sensitivity of 67% (2/3); liver and lung metastases diagnosed by CT were not detected by SSTR-PET (liver 0/5, lung 0/2). Pleural metastases were detected by SSTR-PET in 1 out of 2 patients (Table 2A). Skin metastases were frequently located outside the field-of-view of the CT scan. Hence, a direct comparison was not feasible. Nevertheless, cutaneous metastases were visualized by SSTR-PET in 6 of 8 patients with clinical diagnosis of cutaneous metastases; metastases <5 mm were not detected. Interestingly, several cutaneous metastases did not show increased tracer uptake despite a size >15 mm indicating a reduced sensitivity of SSTR-PET for detection of cutaneous metastases from MCC.

### Diagnostic accuracy of SSTR-PET and CT: lesion-based analysis

Detailed results are given in Table 2B. SSTR-PET detected 62 lymph node metastases as compared to 105 lesions detected by CT (sensitivity, 59%). SSTR-PET revealed focal tracer uptake in all bone and soft tissue lesions detected by CT (sensitivity 100%); moreover, it indicated 15 additional osseous metastases without any correlate at corresponding CT.

SSTR-PET did not show any focal tracer uptake in the liver and the lungs. Inhomogeneity of tracer uptake in liver tissue was noticed but no focal lesions were detected. SSTR-PET detected a single histology-confirmed metastatic lesion in the parotid gland, which was not seen by CT. Another parotid lesion was detected by both SSTR-PET and CT but turned out as benign lesion after histological work-up (Warthin tumor).

### Discordant findings at SSTR-PET and CT

Patient 11 with stage IIIB disease presented with 2 lymph nodes suspected to be metastases by CT while SSTR-PET did not reveal focal tracer uptake. Complete lymph node dissection revealed MCC metastases up to 5 mm in 2 out of 9 nodes. In patient 15 it was contrariwise: CT returned stage IIC disease (N0) whereas SSTR-PET indicated stage IIIB (N1b) (Figure 1); targeted lymph node extirpation confirmed N1a disease. Though, SSTR-PET did not visualize N1b disease detected by CT in one stage IV patient (no. 22). In addition, 3 patients (no. 9, 17, and 20) were found to have subcutaneous in-transit metastases correctly identified by SSTR-PET but not by CT (no. 9, 17 outside field-of-view of the CT scan).

### Change of therapeutic management according to SSTR-PET

Patient-specific changes of management due to SSTR-PET results are listed in Table 1. Changes were observed in 3/24 patients (13%): detection of bone metastases resulted in treatment with bisphosphonates. Moreover, because these patients had disseminated bone lesions, local radiotherapy was excluded from treatment options. Furthermore, change of management could have also been possible in a fourth patient. SSTR-PET positivity of the sentinel lymph node indicated a complete lymph node dissection, but unremarkable morphologic imaging by CT and ultrasound made this lesion doubtful prompting a targeted extirpation instead. Since histologic workup confirmed a metastasis a complete lymph node dissection was performed.

Additional soft tissue or in-transit metastases did not change clinical management since multiple metastatic sites were detected in these patients, hence systemic treatment was initiated.

### Repetitive SSTR-PET

**Figure 3:**
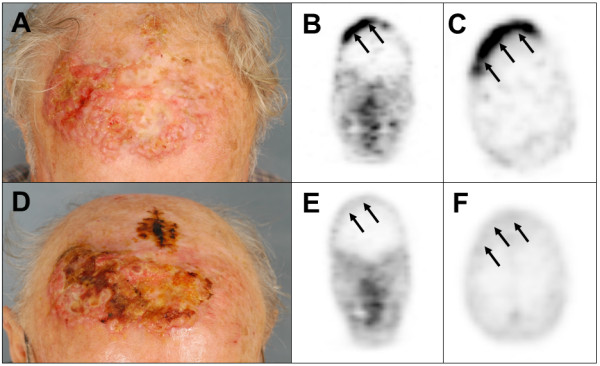
**Repetitive SSTR-PET imaging for assessment of treatment response.** Response to therapy assessed using repetitive SSTR-PET imaging. Patient no. 22 presented with a large primary tumor with satellite metastases **(A)**. The primary tumor showed increased ^68^Ga-DOTATOC uptake **(B, C)**. After combined radio-chemotherapy including liposomal doxorubicin **(D)**, SSTR-expression was no longer detectable by SSTR-PET **(E, F)**.

Eight patients were imaged prior and after chemotherapeutic treatment. Four patients showed no response, neither by SSTR-PET nor by CT (no. 11, 15, 20, and 23). In the remainder, a minor response according to RECIST 1.1 or EORTC PET-criteria [26,27] was indicated by both modalities. Patient no. 14 displayed diminished ^68^Ga-DOTATOC uptakes in the lymph node and bone after initiation of treatment, which is indicative of regression of the disease. In patient no. 16, a mixed response was observed with morphologically smaller, SSTR-PET-negative nodal but progressive pancreatic and new cerebral SSTR-PET-positive lesions. Patient no. 21 had a mixed response with partial remission of liver metastases and progression of lymph node metastases identified by both SSTR-PET and CT. Patient no. 22 showed reduction of cutaneous satellite, and stable nodal as well as bone metastases (Figure 3). However, this short-term response was followed by progression of all metastatic sites and development of new cerebral metastases indicated by SSTR-PET. Of interest, SSTR-PET identified brain metastases during re-staging in 2 patients corresponding to 8% of the total collective, i.e. 20% of the stage IV patients; in one of them, radiotherapy for brain metastases was performed.

## Discussion

In this retrospective study, we have demonstrated that tumor staging using specific imaging of SSTR expression by PET is feasible and provides additional diagnostic information. All primary tumors were visualized with focal uptake of the radiotracer, indicating that SSTR-PET could be used for tumor localization in patients with unknown primary.

SSTR-PET showed higher sensitivity for detection of bone metastases as compared to CT, which is consistent with findings in other neuroendocrine cancers [28]. Notably, diagnosis of bone metastases provided a rationale for initiation of bisphosphonate treatment in those three patients. Interestingly, two patients of our cohort were diagnosed with cerebral metastases based on SSTR-PET. A rate of 8% in the total cohort, i.e. 20% in stage IV patients, is much higher than expected from previous observations that reported central nervous system involvement to be a very rare event in MCC. Thus, SSTR-PET appears as a highly sensitive means to detect cerebral metastases of MCC that may have been under-diagnosed previously [29] and SSTR-PET is superior to FDG-PET in this regard [14]. SSTR-PET was superior to CT regarding detection of soft tissue metastases which could also have further impact on the therapeutic decision making process. Overall, patient management was directly changed in 13% of cases.

In contrast, SSTR-PET turned out to be significantly inferior to CT in terms of imaging liver and lung metastases. This is likely due to physiologically high uptake in the liver. With respect to the lung, pulmonary metastases from MCC are generally small and motion artifacts due to breathing negatively interfere with lesion detection by PET. Detection of cutaneous metastases depends on lesion size. In the present series, lesions had to be at least 5 mm to be visualized with SSTR-PET. However, several skin metastases did not show increased tracer uptake despite a size > 15 mm. A similar result has been recently described for gamma camera-based imaging using ^111^In-octreotide. With this imaging approach none of 14 cutaneous metastases were visualized in 2 patients [30].

**Figure 4:**
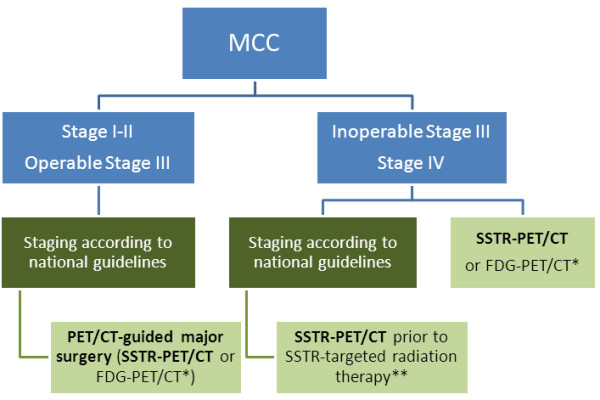
**Proposed staging algorithm for MCC. SSTR-PET/CT should be considered prior to major surgery for stage III disease and for inoperable stage III and stage IV disease.** SSTR-PET/CT should be performed prior to radiopeptide therapy. *Further clinical trials are required comparing DOTATOC/TATE-PET/CT versus FDG-PET/CT. **SSTR-targeted radiation therapy is an experimental treatment for MCC. Further clinical studies are needed.

Lymphatic metastases are frequent in MCC and severely impact prognosis [5]. However, MCC affects the elderly and is frequently localized in the head-and-neck region, thus both SLNB and possible radical lymphadenectomy are associated with an increased surgery risk. In our series, SSTR-PET was true negative in 6 SLN-negative patients and true positive in one SLN-positive patient but failed to demonstrate a 5 mm iliac lymph node metastasis in a patient who underwent complete lymph node dissection. Moreover, since neither SSTR- nor FDG-PET/CT consistently detects nodal MCC micro-metastases, these techniques are not intended to replace the sentinel lymph node biopsy [8,13,15]. However, if SSTR-PET detects unapparent lymph node metastases, as e.g. in patient no. 15, the patient can directly undergo complete lymphadenectomy, i.e. SLNB can be spared. SSTR-PET may have higher diagnostic performance than FDG-PET in MCC as pointed out in a recent case report [24]. In a retrospective analysis, FDG-PET/(CT) has been shown to be a useful tool in management of MCC and may lead to upstaging of 16% of patients [14]. A head-to-head comparison of both imaging approaches (SSTR expression vs. FDG metabolism) warrants an interesting tool for further research to gain deeper knowledge of tumor biology. However, SSTR-PET is superior to FDG-PET for detection of cerebral metastases of MCC and only SSTR-PET harbors the possibility of a theranostic approach towards the disease by combining diagnostic and therapeutic properties. Based on these notions, we propose a staging algorithm, which includes SSTR-PET/CT (Figure 4). Current therapy regimens for metastatic MCC have a limited impact on the overall survival [31]. Recently, treatment with somatostatin analogs has been reported for both MCC and NET [32-36]. In NETs, retention of DOTATOC, as measured by calculation of SUV_mean_ or SUV_max_, is usually at least 2-fold higher as compared to a reference segment in the liver. MCC lesions in our cohort showed markedly lower tracer retention of DOTATOC/-TATE. This fact indicates that response rates to targeted radionuclide therapy (i.e., ^90^Y-DOTATOC or ^177^Lu-DOTATATE) may be inferior, however, MCC is a very radiosensitive tumor and therefore, therapy with radio-labeled somatostatin analogs might nevertheless be considered in an otherwise limited therapeutic scenario.

One of the limitations of our study is immanent to the orphan disease status of MCC: it comprises a limited number of patients; nevertheless, to the best of our knowledge, this is the largest cohort of MCC patients imaged by SSTR-PET to date. Other limitations are also related to the low incidence of MCC: The SSTR-PETs were carried out over a period of 3 years; over this period substantial improvements of SSTR-PET technologies were introduced. Thus, the majority of patients were examined using a dedicated PET scanner and only 5 patients were imaged by PET/CT. Thus, it can be assumed that implementation of integrated PET/CT scanners providing enhanced image quality will facilitate detection of smaller lesions. An example demonstrating improved image quality and sensitivity of PET/CT devices as compared to a PET only device is shown in the Additional file 1: Figure S1. Similarly, the possible benefits of ^68^Ga-DOTATATE over ^68^Ga-DOTATOC had been realized. However, since we could demonstrate the advantage of SSTR-PET in combination with contrast-enhanced CT using older technologies for correct staging of MCC, it can be envisaged that these benefits are even better with improved SSTR-PET techniques.

## Conclusion

This study highlights the role of molecular imaging of SSTR expression in MCC. The advantage of SSTR-PET is based on an improved visualization of bone, soft tissue and brain metastases, whereas limitations relate to an insufficient detection of lung and liver metastases. Therefore, in MCC, SSTR-PET should be always performed in combination with contrast-enhanced CT; indeed, such an approach resulted in a change of patient management in 13% of cases. Furthermore, focally increased tracer uptake suggests treatment of MCC using β-emitter-labeled SSTR analogs such as ^90^Y- or ^177^Lu-DOTATATE as a therapeutic option.

## Competing interests

The authors declare that they have no competing interests.

## Authors’ contributions

KB, CL, MCK, EBB, MG, AKB and JCB are responsible for conception and design. Data were obtained by KB, CL, AlS, and MCK. AS and KH provided technical support. All authors contributed to analysis and interpretation of data, wrote, reviewed and approved the final manuscript.

## Authors’ information

Initials: Kristina Buder (KB), Constantin Lapa (CL), Andreas Schirbel (AS), Ken Herrmann (KH), Michael C. Kreissl (MCK), Alexander Schnack (AlS), Eva Bettina Bröcker (EBB), Matthias Goebeler (MG), Andreas K. Buck (AKB), Jürgen C. Becker (JCB).

## Pre-publication history

The pre-publication history for this paper can be accessed here:

http://www.biomedcentral.com/1471-2407/14/268/prepub

## Supplementary Material

Additional file 1: Figure S1 Improved sensitivity by integrated PET/CT. Example of one patient who received both PET only and contrast-enhanced CT scan **(A-C)** as well as integrated PET/CT imaging six months later **(D-F)**. A small left-sided supraclavicular lymph node (A + D) was depicted as lymph node metastasis by integrated PET/CT indicating focal SSTR expression of the metastatic node (D: fused PET/CT image, E/F: PET). Click here for file

## References

[B1] Schrama D Ugurel S Becker JC Merkel cell carcinoma: recent insights and new treatment options Curr Opin Oncol 2012 24 2 141 149 10.1097/CCO.0b013e32834fc9fe 22234254

[B2] Agelli M Clegg LX Becker JC Rollison DE The etiology and epidemiology of merkel cell carcinoma Curr Probl Cancer 2010 34 1 14 37 10.1016/j.currproblcancer.2010.01.001 20371072

[B3] Durani BK Klein A Henze M Haberkorn U Hartschuh W Somatostatin analogue scintigraphy in Merkel cell tumours Br J Dermatol 2003 148 6 1135 1140 10.1046/j.1365-2133.2003.05338.x 12828740

[B4] Fantini F Johansson O Neurochemical markers in human cutaneous Merkel cells. An immunohistochemical investigation Exp Dermatol 1995 4 6 365 371 10.1111/j.1600-0625.1995.tb00061.x 8608344

[B5] Lemos BD Storer BE Iyer JG Phillips JL Bichakjian CK Fang LC Johnson TM Liegeois-Kwon NJ Otley CC Paulson KG Ross MI Yu SS Zeitouni NC Byrd DR Sondak VK Gershenwald JE Sober AJ Nghiem P Pathologic nodal evaluation improves prognostic accuracy in Merkel cell carcinoma: analysis of 5823 cases as the basis of the first consensus staging system J Am Acad Dermatol 2010 63 5 751 761 10.1016/j.jaad.2010.02.056 20646783 PMC2956767

[B6] Becker JC Assaf C Vordermark D Reske SN Hense J Dettenborn T Seitz O Grabbe S German guideline Merkel Cell Carcinoma JDDG 2013 Suppl. 3 31 38 10.1111/ddg.12015_6 23734895

[B7] Boccara O Girard C Mortier L Bens G Saiag P Guillot B Guidelines for the diagnosis and treatment of Merkel cell carcinoma - Cutaneous Oncology Group of the French Society of Dermatology Eur J Dermatol 2012 22 3 375 379 22498750 10.1684/ejd.2012.1694

[B8] Colgan MB Tarantola TI Weaver AL Wiseman GA Roenigk RK Brewer JD Otley CC The predictive value of imaging studies in evaluating regional lymph node involvement in Merkel cell carcinoma J Am Acad Dermatol 2012 67 6 1250 1256 10.1016/j.jaad.2012.03.018 22552001

[B9] Sarnaik AA Zager JS Cox LE Ochoa TM Messina JL Sondak VK Routine omission of sentinel lymph node biopsy for merkel cell carcinoma < = 1 cm is not justified J Clin Oncol 2010 28 1 e7 10.1200/JCO.2009.25.9937 19933899

[B10] Schwartz JL Griffith KA Lowe L Wong SL McLean SA Fullen DR Lao CD Hayman JA Bradford CR Rees RS Johnson TM Bichakjian CK Features predicting sentinel lymph node positivity in Merkel cell carcinoma J Clin Oncol 2011 29 8 1036 1041 10.1200/JCO.2010.33.4136 21300936 PMC5321088

[B11] Stokes JB Graw KS Dengel LT Swenson BR Bauer TW Slingluff CL Jr Ledesma EJ Patients with Merkel cell carcinoma tumors < or = 1.0 cm in diameter are unlikely to harbor regional lymph node metastasis J Clin Oncol 2009 27 23 3772 3777 10.1200/JCO.2008.20.8272 19581538 PMC2727285

[B12] Belhocine T Pierard GE Fruhling J Letesson G Bolle S Hustinx R Dargent JL Flamen P Rigo P Clinical added-value of 18FDG PET in neuroendocrine-merkel cell carcinoma Oncol Rep 2006 16 2 347 352 16820914

[B13] Concannon R Larcos GS Veness M The impact of (18)F-FDG PET-CT scanning for staging and management of Merkel cell carcinoma: results from Westmead Hospital, Sydney, Australia J Am Acad Dermatol 2010 62 1 76 84 10.1016/j.jaad.2009.06.021 20082888

[B14] Hawryluk EB O’Regan KN Sheehy N Guo Y Dorosario A Sakellis CG Jacene HA Wang LC Positron emission tomography/computed tomography imaging in Merkel cell carcinoma: a study of 270 scans in 97 patients at the Dana-Farber/Brigham and women’s cancer center J Am Acad Dermatol 2013 68 4 592 599 10.1016/j.jaad.2012.08.042 23127473

[B15] Maury G Dereure O Du-Thanh A Mariano-Goulart D Guillot B Interest of (18)F-FDG PET-CT scanning for staging and management of merkel cell carcinoma: a retrospective study of 15 patients J Eur Acad Dermatol Venereol 2011 25 12 1420 1427 10.1111/j.1468-3083.2011.03994.x 21366705

[B16] Peloschek P Novotny C Mueller-Mang C Weber M Sailer J Dawid M Czerny C Dudczak R Kletter K Becherer A Diagnostic imaging in Merkel cell carcinoma: lessons to learn from 16 cases with correlation of sonography, CT, MRI and PET Eur J Radiol 2010 73 2 317 323 10.1016/j.ejrad.2008.10.032 19108971

[B17] Siva S Byrne K Seel M Bressel M Jacobs D Callahan J Laing J Macmanus MP Hicks RJ 18 F-FDG PET provides high-impact and powerful prognostic stratification in the staging of Merkel cell carcinoma: a 15-year institutional experience J Nucl Med 2013 54 8 1223 1229 10.2967/jnumed.112.116814 23753187

[B18] Kwekkeboom DJ Hoff AM Lamberts SW Oei HY Krenning EP Somatostatin analogue scintigraphy. A simple and sensitive method for the in vivo visualization of Merkel cell tumors and their metastases Arch Dermatol 1992 128 6 818 821 10.1001/archderm.1992.01680160102014 1599271

[B19] Hofmann M Maecke H Borner R Weckesser E Schoffski P Oei L Schumacher J Henze M Heppeler A Meyer J Knapp H Biokinetics and imaging with the somatostatin receptor PET radioligand (68)Ga-DOTATOC: preliminary data Eur J Nucl Med 2001 28 12 1751 1757 10.1007/s002590100639 11734911

[B20] Boy C Heusner TA Poeppel TD Redmann-Bischofs A Unger N Jentzen W Brandau W Mann K Antoch G Bockisch A Petersenn S 68Ga-DOTATOC PET/CT and somatostatin receptor (sst1-sst5) expression in normal human tissue: correlation of sst2 mRNA and SUVmax Eur J Nucl Med Mol Imaging 2011 38 7 1224 1236 10.1007/s00259-011-1760-x 21369945

[B21] Miederer M Seidl S Buck A Scheidhauer K Wester HJ Schwaiger M Perren A Correlation of immunohistopathological expression of somatostatin receptor 2 with standardised uptake values in 68Ga-DOTATOC PET/CT Eur J Nucl Med Mol Imaging 2009 36 1 48 52 10.1007/s00259-008-0944-5 18807033

[B22] Gabriel M Decristoforo C Kendler D Dobrozemsky G Heute D Uprimny C Kovacs P von Guggenberg E Bale R Virgolini IJ 68Ga-DOTA-Tyr3-octreotide PET in neuroendocrine tumors: comparison with somatostatin receptor scintigraphy and CT J Nucl Med 2007 48 4 508 518 10.2967/jnumed.106.035667 17401086

[B23] Schneider C Schlaak M Bludau M Markiefka B Schmidt MC 68Ga-DOTATATE-PET/CT positive metastatic lymph node in a 69-year-old woman with Merkel cell carcinoma Clin Nucl Med 2012 37 11 1108 1111 10.1097/RLU.0b013e318266d3b3 22996252

[B24] Epstude M Tornquist K Riklin C di Lenardo F Winterhalder R Hug U Strobel K Comparison of 18 F-FDG PET/CT and 68Ga-DOTATATE PET/CT imaging in Metastasized Merkel cell carcinoma Clin Nucl Med 2013 38 4 283 284 10.1097/RLU.0b013e318281658e 23429397

[B25] Breeman WA de Blois E Sze Chan H Konijnenberg M Kwekkeboom DJ Krenning EP (68)Ga-labeled DOTA-peptides and (68)Ga-labeled radiopharmaceuticals for positron emission tomography: current status of research, clinical applications, and future perspectives Semin Nucl Med 2011 41 4 314 321 10.1053/j.semnuclmed.2011.02.001 21624565

[B26] Eisenhauer EA Therasse P Bogaerts J Schwartz LH Sargent D Ford R Dancey J Arbuck S Gwyther S Mooney M Rubinstein L Shankar L Dodd L Kaplan R Lacombe D Verweij J New response evaluation criteria in solid tumours: revised RECIST guideline (version 1.1) Eur J Cancer 2009 45 2 228 247 10.1016/j.ejca.2008.10.026 19097774

[B27] Young H Baum R Cremerius U Herholz K Hoekstra O Lammertsma AA Pruim J Price P Canc EORT Measurement of clinical and subclinical tumour response using [F-18]-fluorodeoxyglucose and positron emission tomography: review and 1999 EORTC recommendations Eur J Cancer 1999 35 13 1773 1782 10.1016/S0959-8049(99)00229-4 10673991

[B28] Putzer D Gabriel M Henninger B Kendler D Uprimny C Dobrozemsky G Decristoforo C Bale RJ Jaschke W Virgolini IJ Bone metastases in patients with neuroendocrine tumor: 68Ga-DOTA-Tyr3-octreotide PET in comparison to CT and bone scintigraphy J Nucl Med 2009 50 8 1214 1221 10.2967/jnumed.108.060236 19617343

[B29] Bailey TL Fung MA Gandour-Edwards R Ellis WG Schrot RJ Clinical emergence of neurometastatic merkel cell carcinoma: a surgical case series and literature review J Neurooncol 2011 102 1 147 155 10.1007/s11060-010-0304-8 20668913 PMC3041920

[B30] Guitera-Rovel P Lumbroso J Gautier-Gougis MS Spatz A Mercier S Margulis A Mamelle G Kolb F Lartigau E Avril MF Indium-111 octreotide scintigraphy of Merkel cell carcinomas and their metastases Ann Oncol 2001 12 6 807 811 10.1023/A:1011142410535 11484956

[B31] Voog E Biron P Martin JP Blay JY Chemotherapy for patients with locally advanced or metastatic Merkel cell carcinoma Cancer 1999 85 12 2589 2595 10.1002/(SICI)1097-0142(19990615)85:12<2589::AID-CNCR15>3.0.CO;2-F 10375107

[B32] di Bartolomeo M Bajetta E Buzzoni R Mariani L Carnaghi C Somma L Zilembo N di Leo A Clinical efficacy of octreotide in the treatment of metastatic neuroendocrine tumors. A study by the Italian trials in medical oncology group Cancer 1996 77 2 402 408 10.1002/(SICI)1097-0142(19960115)77:2<402::AID-CNCR25>3.0.CO;2-4 8625251

[B33] Fakiha M Letertre P Vuillez JP Lebeau J Remission of Merkel cell tumor after somatostatin analog treatment J Cancer Res Ther 2010 6 3 382 384 10.4103/0973-1482.73352 21119285

[B34] Meier G Waldherr C Herrmann R Maecke H Mueller-Brand J Pless M Successful targeted radiotherapy with 90Y-DOTATOC in a patient with Merkel cell carcinoma. a case report Oncology 2004 66 2 160 163 10.1159/000077443 15138369

[B35] Salavati A Prasad V Schneider CP Herbst R Baum RP Peptide receptor radionuclide therapy of Merkel cell carcinoma using (177)lutetium-labeled somatostatin analogs in combination with radiosensitizing chemotherapy: a potential novel treatment based on molecular pathology Ann Nucl Med 2012 26 4 365 369 10.1007/s12149-012-0578-3 22361935

[B36] Schmidt MC Uhrhan K Markiefka B Hasselbring L Schlaak M Cremer B Kunze S Baum RP Dietlein M (68)Ga-DotaTATE PET-CT followed by peptide receptor radiotherapy in combination with capecitabine in two patients with merkel cell carcinoma Int J Clin Exp Med 2012 5 4 363 366 23293710 PMC3526339

