# Supplementary figures and images for: Somatostatin receptor expression in Merkel cell carcinoma as target for molecular imaging
Source: BMC Cancer. 2014 Apr 17;14:268. doi: 10.1186/1471-2407-14-268 (PMC4021101; doi:10.1186/1471-2407-14-268)

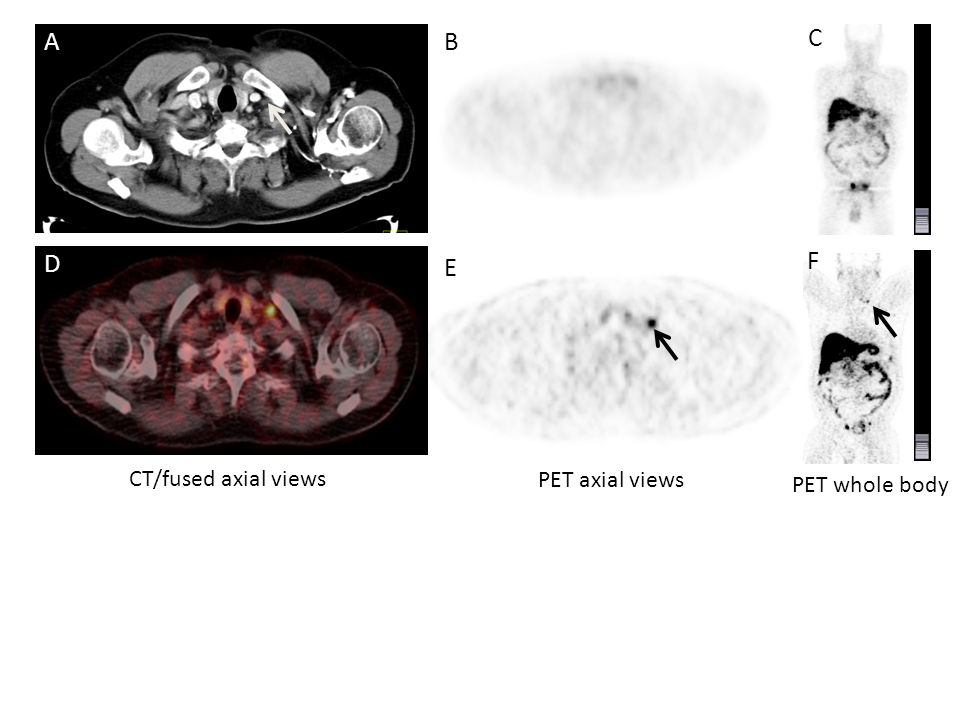

Supplement: Additional file 1: Figure S1 — Improved sensitivity by integrated PET/CT. Example of one patient who received both PET only and contrast-enhanced CT scan (A-C) as well as integrated PET/CT imaging six months later (D-F). A small left-sided supraclavicular lymph node (A + D) was depicted as lymph node metastasis by integrated PET/CT indicating focal SSTR expression of the metastatic node (D: fused PET/CT image, E/F: PET). [file 1471-2407-14-268-S1.tiff]
